# Organisational and leadership skills towards healthy workplaces: an interview study with registered nurses in Sweden

**DOI:** 10.1186/s12912-024-01732-3

**Published:** 2024-01-19

**Authors:** Kristina Rosengren, Malin Friberg

**Affiliations:** 1 Institute of Health and Care Sciences, Sahlgrenska Academy, University of Gothenburg, Gothenburg, SE-405 30 Sweden; 2 Centre for Person-centred Care (GPCC), Sahlgrenska Academy, University of Gothenburg, Gothenburg, SE-405 30 Sweden; 3 Department of Internal Medicine, Sahlgrenska University Hospital, Mölndal, Sweden

**Keywords:** Person-centred approach, Qualitative content analysis, Registered nurses, Sweden, University hospital, Working environment

## Abstract

**Background:**

According to shortage of registered nurses, organisational and leadership aspects grounded in person-centrered approach, are highlighted to ensure high quality of care. Therefore, it is interesting to develop knowledge regarding registered nurses working environment.

**Aim:**

The aim of the study was to investigate registered nurses’ reason to end their employment at a university hospital setting (internal medicine, emergency department).

**Method:**

Qualitative content analysis with an inductive methodological approach was used to analyse registered nurses’ experiences regarding their former employment. Inclusion criteria; all nurses (*n* = 55) who ended employment during one year (first of July 2020-30th of June 2021) were invited, and 38 semi-structured interviews were conducted.

**Results:**

Three categories were identified: Limited organisational support, Lack of visible leadership, and Limited healthy working environment, followed by six subcategories: Longing for organisational support, Being a tile in a box, Need for professional relationship, Limitation of supportive leadership, Imbalance of work versus personal life, and Ethical stress.

**Conclusion:**

To improve registered nurses working environment and commitment to work, balance between time at work and personal life is significant. Therefore, organisational support and leadership skills grounded in a person-centred approach are crucial to develop a healthy working environment. A person-centred leadership could improve collaboration and shared decision-making in partnership with those involved, managers, nurses, and team members.

## Background

Research [1, 2] has shown that high workload, irregular working hours, and a stressful and insecure work environment are reasons that registered nurses (RN) leave their workplace. The above reasons result in a shortage of RN, which negatively influences the work environment for the remaining staff members and quality of care they provide. A lack of RN has put lights on the significant of provide good work conditions for all staff members. Limited working environment is one reason, why improvement is stressed such as interpersonal support, so called nourishment, between colleagues, for example that physicians respect and trust RN’s daily work as well as progress in solving more and more complex challenges in daily work independently and/or teamwork with RN’s and other health professionals [1]. When health professionals accept goals and values within their organisation, organisational commitment are developed, a force to remain working at the workplace [2].

Research [3] highlight that organisational commitment was significant for RN’s staying in nursing (β = 0.32, *p* = .000). For example, burnout and job satisfaction had a significant relationship for RN to staying in nursing (ITSN). Psychosocial factors, for example limited staffing and conflict management, caused by increased administrative tasks among first-line managers, affect work stress and ill health at the workplace, and why both RN’s and managers’ workload are important to manage [4–6]. Committed working conditions is required to create supporting caring environment including well planned information structure for patients, relatives, and staff [7], therefore perceived organizational support is highlighted to create sustainable workplaces for RN’s [8]. A well-functioning organisation structure with specific, measurable, agreed, realistic and time-specific goals give possibilities for team members to take response for the care units tasks [4, 9]. Moreover, staff with access to structural empowerment have present and available managers [7], therefore, it is significant that staff had access to structural empowerment especially when managers work continually and intentionally with a lot of tasks at the same time [6]. Leadership based on relationship and partnership provide conditions for sustainable working life, challenging tasks within a turbulent care setting with high workload and staff turnover [6, 10]. On example is shared leadership among team members in collaboration with shared decision-making tasks towards high quality of care [11].

Person-centred care could be described that person’s is more than her/his disease, it is a person with resources, abilities and needs. Therefore, person-centred approach is based on capable persons (resources, abilities) who in relation to others (teamwork) improve healthcare (evidence, proven experience) by supporting healthy caring/working environment (security, continuity) for both patients, relatives, employees, and managers. Partnerships are based on narratives from actors involved (staff, managers, patients, relatives) focusing resources/abilities which are documented in different kind of agreements through shared decision-making which strengthen teamwork among those involved [4, 12, 13]. The importance of systematic work due to person-centred approach is highlighted to contribute to efficiency in healthcare due to RN’s working environment and patient safety [9, 14, 15]. To equip healthcare organizations of the future with increasing caring needs towards limited resources (monetary, personnel), person-centred care (PCC) and person-centred working methods (PCW) are highlighted for shorter care events, satisfied patients, fewer readmissions, higher quality of care, better work environment and better resource utilization [16, 17]. To our knowledge there is limited knowledge why RN’s choose to change workplaces, therefore, the aim of the study was to investigate registered nurses reason to end their employment at a university hospital setting (internal medicine, emergency department).

## Methods

### Settings

The study used a convenience selection of one Sweden’s largest university hospital with strong innovation and clinic research in close and broad cooperation with academia, industry and patients, with skills to carry out the advanced treatment to ensure high standard of quality in close to all areas of education and research. The hospital is located in western part of Sweden with approximately 17 000 employees divided into 120 departments located at four different hospitals, current study was conducted at one of these hospitals included emergency department, intensive care unit (ICU), operating theatre, wards (mostly medicine, orthopaedic, geriatric) as well as day care units. Data were collected from former staff members who had worked within internal medicine and/or emergency department.

### Design

This study used qualitative content analysis [18, 19], a qualitative method, to increase the understanding of RN’s voices, views, and thoughts about their former work that they choose to end in the year 2020 [20]. Qualitative content analysis [18, 19] illustrates use of several concepts that relate to research procedures answering the study aim, to investigate why RN choose to end their employment at a university hospital setting towards trustworthiness, credibility, dependability, and transferability. Qualitative research design is “information driven” and can be helpful developing insightful and appropriate interpretations within healthcare. An inductive methodological approach [20] was used to analyse data based on the content of RN’s thoughts and experiences regarding their former employment in nursing.

### Data collection

A convenience sample was used, which are described as appropriate for qualitative methods [20] such as content analysis [18, 19]. The inclusion criteria was for participation were registered nurses (+ 18 years), minimum six-month experiences of healthcare at the specific university hospital (internal medicine, emergency department) with ability to understand and speak Swedish. The rational why the study focusing above university hospital and specific units was the impact of COVID-19 pandemic on health human resources at the specific healthcare setting where the authors work. Exclusion criteria were health professionals employed at other hospitals. Permission for the study were obtained from the managers of included department at the university hospital and human relations department. All RN (*n* = 55) who ended their employment during one year (first of July 2020-30th of June 2021) were invited (phone calls, text messages) to participate by two staff members at the Human Relation department. Participants were informed (oral, written information) about the study aim, method and how forthcoming results were going to be used due to voluntary and confidential participation. One reminder (text messages) was sent out, and all interested RN were included (*n* = 38) after that informed consent was collected in accordance with the Declaration of Helsinki [21], such as given information about study aim, methods and use of forthcoming results. Informed Consent was deemed unnecessary (national regulations) [22] when conducting quality improvement (health professionals). However, all methods were carried out in accordance with relevant guidelines and regulations, both national and international [18–23]. Respect for individual was a main concern during the study, no names were used in quotes, and the results are described in categories (group level). Respect for the participants’ integrity and autonomy was thereby shown throughout the study [21, 23].

The data were collected in May to September 2021 by telephone interviews, however one interview was conducted as video conference. All interviews started with background questions, including questions regarding age, education, and personal experience with healthcare. Furthermore, data collection focused on seven perspectives: reason for ending employment, consideration of re-employment, impact of Covid-19, managerial aspects, workload, new employment, improvements, and future aspects. The interviews started with “Could you describe the background to your decision to end your employment at the hospital?” Based on the answers, related questions were asked regarding above seven perspectives. Examples of situations such as positive and negative aspects of work at the hospital were explored, and clarifications and further elaborations were made. The interviews lasted between 20 and 40 min and were performed individually by two interviewers working in the Human Relation department without professional relationship to participants former work at the hospital. Thereafter, all interviews were transcribed verbatim directly when the interview were finished. Participants (*n* = 38) were registered nurses ages 21–64 (md = 42 year) experience healthcare between 2 and 42 year (md = 15 year).

### Data analysis

The interviews were analysed by using manifest qualitative content analysis [18, 19] to interpret meaning from data to address trustworthiness, with examples drawn from the area of RN’s experience of working within the hospital settings (internal medicine wards, emergency department). Data analyses were grounded in well used scientific methods focusing what content participants talk about, a close approach and concrete analysis level, close to participants experiences, being innate, acquired and/or socially constructed. Text from interviews was divided into meaning units, condensed and coded (differences, similarities), i.e. a process of reflection and discussion until authors agreed on categories and subcategories. Hereby is a description of our data analysis. Written words were basis for data analysis, which was performed in following steps (1–5) and examples of the analysis process is presented in Table 1. First (1), the transcripts were read and re-read to obtain an understanding of and familiarity with data; Second (2), meaning units (words, sentences or paragraphs) that corresponded to the content areas in line with the aim were selected by using an inductive approach concerning (a) high workload and (b) lack of support; Thereafter (3), each meaning unit was condensed into a description of its content and labelled with one of 43 codes for example stressful working environment. All categories were identified and grouped related to the codes (4); and at last (5), three categories was identified (Limited organizational support, Lack of visible leadership and Limited healthy working environment), followed by six subcategories (Longing for organizational support and Tile in a box; Professional relationship and Supportive leadership; Imbalance work versus private life and Ethical stress) responding to why RN choose to end their employment at the university, illustrated with quotes [20].

**Table 1:** Examples of the analysis processes

Meaning unit	Condensation	Code	Subcategory	Category
…no leisure time, especially when we have to work every other weekend. The day off duty, I was so tired I just slept the whole day	no leisure time, work every other weekend day off duty, tired, just slept whole day	Stressful working environment	Imbalance work versus private life	Limited healthy working environment
…I understand that it is hard even for first line manager, but there is room for improvement as better dialogues between me and my manager…	…understand hard for first line managers, room improvement, better dialogue	Priorities work tasks	Professional relationship	Lack of visible leadership
I am disappointed in how I was treated at work	Disappointed treated at work	Interchangeable as registered nurses	Tile in a game	Limited organizational support

## Results

**Fig. 1:**
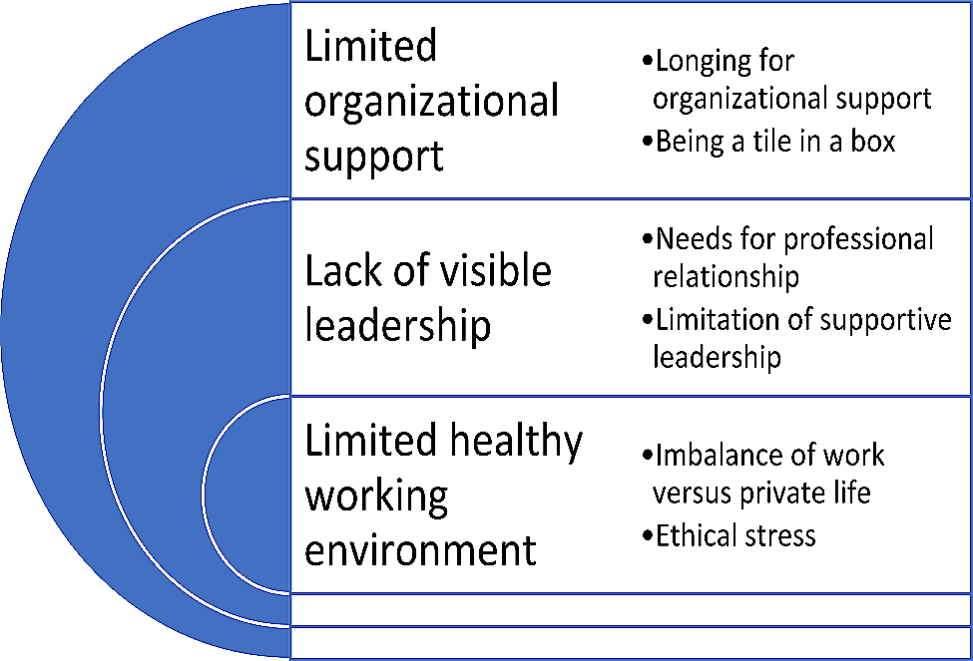
Overview of categories and subcategories

The results are presented by three categories; Limited organizational support, Lack of visible leadership and Limited healthy working environment, followed by six subcategories; Longing for organizational support and Being a tile in a box; Need for professional relationship and Limitation of supportive leadership; and finally Imbalance of work versus private life and Ethical stress (Fig. 1).

### Limited organizational support

The category limited organizational support include following content such as longing for organizational support to manage day to day work at the care unit, and why feeling such as being a tile in a box, without dignity and respect as health professionals who easily could be replaced with another RN’s, was described.

### Longing for organizational support

The subcategory longing for organizational support includes limited introduction of newly employed RN’s (newly educated, experienced RN) and/or late schedule of duty at the care unit. Limited organizational support decrease RN’s day to day burden such as repeated introduction and supervision of new staff members occurred randomly without coordination at different levels (care unit, department, hospital). Participants stress that organizational support was needed due to recruitment and introduction of new staff members, for example lack of systematic planning at the hospital level occurred on regularly basis when new staff members was needed. This were expressed as;

*There has been a high expectation on us that has worked the longest. A lot of demand on teaching that takes energy very much. That leads to that I feel insufficient because I haven’t got time for it all. Not enough time to help all the patient. I feel taken advantage of and sad. In addition to being understaffed according to me we are expected to swap rotes end annual leave which we can´t do for any length of time*.

Shortage of RN’s negatively influenced day to day work though introduction of continuously number of new staff members were added to RN (experience, less experienced) who already had a high workload in day-to-day work. Participants points out that introduction was unstructured, unplanned, and too short due to lack of systematic organizational support.

*A bit longer introduction could maybe have made him/her like it more, it is hard the first time, too much responsibility. You are the nurse and you are the one that should solve it, that kind of attitude, a hard feeling when you are new and insecure.*

Therefore, RN highlight improvements such as both standardized introductions, according to general hospital structure, routines and tasks supported from human relation department as well as unit specific introduction supported by the team at the workplace.

Low salary was another reason highlighted by the participants as reason to their leave work at the hospital. RN stressed that their first line managers supported higher RN salary, however raising salary were not authorized from the hospital managerial level. They argued that limited organizational governance increases the shortage of RN at the university hospital. Moreover, Covid-19 was described as interesting phenomena due to severe illness, which develop participants knowledge and skills to do what RNs are educated for; nursing, and why RNs prolonged their employment instead of early ending. However, RNs point out improvement such as better planning of staffs schedule according to the pandemic, though they experienced limited planning when shift were rapidly and repeatedly changed resulted in limited patient safety at the care unit. Unstructured and late change of RN’s duty schedule was caused by limited organizational support at the care unit. RN stress several changes of work schedule was without enough time to prepare for duty. This resulted in uncertainty regarding how future wok schedule would look like why planning for their private life was difficult. Unstructured work schedule negatively influences work satisfaction and why participant choose to end their employment. Therefore, shortage of RN continuing though colleagues choose to end the employment at the care unit, i.e., moment 22.

### Being a tile in a box

The subcategory being a tile in a box include staffing problem and limited duty planning at the care unit, and why feelings being replaceable without notice of knowledgably unique person was described.

*The feeling that the managers hired and was satisfied if they only got a name on the list. It was not safe for the patients*.

Participants stress that lack of available RN on duty at the care unit caused uncertainty among employed RNs. Lack of RN affected participants possibilities of planning their leisure time and why uncertainty occurred due to unstructured private life. Uncertainty regarding if and when they could be on duty caused feelings as being a tile in a box, being “only” a RN, not a person who are skilled in nursing.

*Feel like the management/leadership was very stressed och pressed, did not get any support despite it being tuff. Unclear directives, small amount of support. Did not think that they handled it well. Felt like a marker in a game, did not see to the individual. Was not visible for the managers” You were just somebody they could play around with*.

Participants stress that this organizational uncertainty due to shortage of RNs decrease feeling of being a tile in a box without respect as a unique person with a private life affect all RN at the workplace.

Another organizational aspect was that information at the employment interview was not fulfilled after they was employed, and why expectations was unmet among newly employed staff members. For example, presentation of the care unit goal and vision was not in line with what participants experiences when she started to work. Moreover, participants also described feeling of being neglected when the workplace was experiences as unfair, such as separate staff members got advantages at work due to personal relationship instead of grounded in organizational aspects. In addition, work schedules during weekends were permanently changed, from three out of five to two out of four weekends (every other weekend), which negatively influenced participants private life with less available time for families and friends.

*No spare time, especially not when we started working every other weekend. On my free time I slept because I was so tired. There was no balance, my personal life went down completely, my partner wanted to leave me because I was only at work and could only think of work.*

### Lack of visible leadership

The category lack of visible leadership include content such as professional relationship and how to improve a supportive leadership. Participants described limited knowledge regarding how and what was included in the management work, unknowing who, what and how the managerial level was conducted due to lack of visible leadership.

### Needs for professional relationship

The subcategory needs for professional relationship include the significant of fair and professional leadership built upon workplaces common tasks to be fulfilled together by all staff members at the care unit. Participants highlight the importance of fair managers who put the ward together, patient first in cooperation with all staff without personal benefits to a separate staff member. Participants stress that first line managers try to do their very best, however organizational limitations such as ongoing recruitment of both managers and staff members was barriers to professional relationship.

*It was a change of managers a couple of times and I felt like we weren’t listened to by the management*.

Managers was busy of day-to-day recruitment which negatively affect the possibilities of professional development due to nursing. RNs points out that visible and committed leadership need to be grounded in day-to-day activities, independently of personal or private relationship within the staff group.

*A lot of new staff so the leadership is necessary due to no real structure on the ward, a lot has been lacking in the leadership for example lack of support*.

According to limited professional support from their managers caused by high workload among managers, mostly from their first line managers, negatively influenced a non-visible manager at the department level as well as at the hospital level (meso level).

*To be listened to and recognized for the work you do was lacking from the leadership*.

Participants described uncertainty what managers doing on daily basis such as what kind of tasks they are dealing with, and why they call for clarity in what management included and how it affects RNs daily work at the care unit. Therefore, managerial visibility at different levels (micro, meso) was highlighted to solve common tasks in collaboration with different hospital units such as emergency department, intensive care unit, wards etcetera.

### Limitation of supportive leadership

The subcategory limitation of supportive leadership includes contents such as support to staff members and fair decision-making at the care unit. Supportive leadership was stress as a tool when routines was changed. However, participants described lack of or limited leadership especially during Covid-19 with several changes due to developed knowledge and skills during the pandemic. According to invisible manager at the care unit, sometimes for several days, participants lack feedback and day-to day leadership, and why insignificant and double work was described, especially among less experienced RN. Limited leadership negatively influenced communication channels and why missing information was highlighted. Roomers occurred due to lack of information, and why false information was used as explanation of implemented changes. Therefore, changes were conducted without explanation that caused frustration and worries among staff.

*I regrated that I started work at XXX due to poor management. I liked my former workplace but wanted to try something new which did not turn out like I had imaged it. There was others that feel the same thing, poor management which led to an unpleasant environment at xxx*.

Negative emotions were connected to experiences as not being listened to when work environment was discussed at the workplace. Lack of appreciation in daily work was pointed out as an important leadership task to fulfil, and why RN felt invisible without any value to the hospitals vision and fulfilment. Participants experienced themselves as a burden to the care unit without possibilities for professional growth, why they apply for another work.

### Limited healthy working environment

The category limited healthy working environment include imbalance of work versus private life, which negatively influence ethical stress in nursing. Participants described high workload due to high number of severe ill patients as well as changed working schedule as well as limited introduction of new staff members.

### Imbalance of work versus private life

The subcategory imbalance of work versus private life included contents as constant fatigue and lack of recovery due to high workload. Participants describe that shortage of RN increase the workload regarding lack of available staff and why they must cover up in nursing on daily basis. This amount of work had impact on their private life though they always were tired without possibility to recovery with work always in their minds, even when they have left work or had a day of. This imbalance was described as;

*What disturbed me and was stressful was that even when I had a weekend of I knew in the back of my mind that work could call me. The weekends I was working it was in the back of my mind that I should have to stay if someone on the next shift was ill. It was stressful that we nurse and nurse assistants had the responsibility to call in someone if anybody was ill to care for the patients. It resulted in that you became unfocused on the patients to solve a sick leave. I did not that, that was ok, and it created stress.*

Participants stressed lack of interest, at the organizational and managerial (hospital, meso) level, when they tried to describe their view of RN’s day to day work. They point out several reasons for RN’s high workload such as high number of incoming severe ill patients, a lot of them with illness due to Covid-19, lack of introduction of newly employed RN (unplanned, to short) and complexed nursing tasks (internal medicine, emergency care). RN high workload had negative impact balancing working and private life, and why emotions of frustration and anger occurred. Participants points out that they were “trapped” within an unhealthy working environment towards with a never ending to do list, and why they felt uninspired and lack of job satisfaction at the end of the working day. This situation was put on repeat, which caused frustration though the same working situation occurred the next day without possibilities to improve it. Therefore, participants point out that stressful working environment was grounded in high demands (organisational, individual) of deliver high quality of care in combination with complexed nursing task to solve. High demands involved emergency medical procedures towards high quality of internal medical care in a stressful working environment (large number of severe ill patient/RN). Low level of control was described from a hospital perspective; single RN cannot improve number of available beds, number of patient per RN as well as routines at the workplace and/or at the hospital. Above high demands in combination with low level of control at work caused negative influence on RN’s working environment. Participants stressed that they did not find possibility to improve a healthy working environment, they called it mission impossible why searching for another healthier workplace were made.

### Ethical stress

The subcategory ethical stress included contents as worries for patient safety as well as lack of satisfaction in relation to RNs ethical code, to do well without harm those patients involved. Participants highlight high ethical stress not doable in the long run, therefore they choose to leave for another workplace less stressful than the former workplace, to still be healthy as a RN to fulfil RNs ethical code, do no harm by improve health and well-being to patients and relatives.

*To be distrusted when we say that we need competence, and nothing happens and also how many we need is very frustrating and stressful. Felt like talking to deaf ears. To work when it doesn’t feel safe for the patients creates an incredibly stress*.

Participants argue that is not possible to stay healthy when their work always remind them of what you forgot or did not have time to do before your shift ended. There highlighted that there were always tasks to fulfil, a never-ending story which create inner ethical stress among RN. This ethical stress was described as being absent in nursing (though, action) without possibility for them as RN to recharge their mind with no possible to leave nursing behind when they end working (day-, evening-, night shift). One reason why they keep staying at the care unit for work was nice and helpful nursing colleagues that helped them to cope with ethical stress at work. However, in the end it was not enough with nice colleagues, they stop their employment searching for a healthier working environment to fulfil RN’s ethical code, to do good without harm.

Moreover, participants stress that all RN (less experienced, experienced RN) continuously introduce new colleagues, mostly newly educated in nursing, which increase ethical stress especially among less experienced RN who felt new by themselves, i.e., double novice in nursing as well as routines at the care unit.

*Well, of course it has been diffucult, a lot of pressure on you. If you are relatively new by yourself and have a lot of people next to you every other day. It created both internal and ethical stress, I felt divided.*

Introduction was also described as an ethical burden for experienced and skilled RN who had to cover up in nursing due to large number of new and novice nursing colleagues. Overall, introduction of new colleagues create ethical stress for all involved though repeated introduction were conducted in limited time (hours) and content (nursing, routines), and at the same time that ordinary nursing activities need to managed, i.e., double burden. Therefore, both experienced (patient safety), less experienced RN (unsure nursing, routines) and newly employed RN (novice routines) stress high workload. According to shortage of RN, all was affected which resulted in high workload for all involved in different perspectives.

## Discussion

The aim of the study, to investigate registered nurses’ reason to end their employment at a university hospital setting (internal medicine, emergency department), was achieved. The results focusing organizational aspects such as lack of organizational support which negatively affected RN’s possibilities to manage day to day work at the care unit, which influence feelings as being a tile in a box without dignity and respect, a person who easily could be replaced with another RN. Another point of view described in the results was that RN longing for visible leadership including professional relationship and supportive leadership. RN’s described limited knowledge regarding management work unknowing who, what and how different managerial level (micro, meso) was conducted due to limited visible leadership at their workplace. The last part of current study describes limited healthy working environment causing imbalance of work (high workload) versus private life causing ethical stress in nursing. RNs high workload was grounded in high number of severe ill patients, changed working schedule (every other weekend, extra duty) as well as limited introduction of continually high number of new staff members due to shortage of RN. Above experiences were reasons for RN to leave their employment at the university hospital for another, hopefully, healthier workplace.

Motivation, trust and respect at work are significant factors towards a healthy working environment [1]. Therefore, continuous learning during daily work is highlighted grounded in interpersonal support between colleagues as well as perceived organizational support is important to improve sustainable workplaces [8]. Nursing preceptorship, a supportive and reflective approach, is one suggestion to promote a healthy working environment by manage complex challenges in everyday nursing at internal medicine wards with high number of severe ill patients and shortage of RN [24]. Expert RN with specific task as tutors supports less experienced RN in daily work to create patient safety with less ethical stress due to respect and trust in RN’s daily work. Nursing preceptorship is in line with a description of catalysts [1] that facilitate independently work along with opportunity to work together with other registered nurses. Nursing preceptorship could improve RN limited healthy working environment, i.e. facilitate a safer workplace culture by advocate RN to easily voice their concerns in day-to-day situation offering a communication area, connecting RN to the overall work at the specific workplace for example general items regarding working environment in various meeting agendas (professional, ward specific) [1, 24, 25]. Support at daily basis is one way to balance work versus private life as well as decrease RN ethical stress.

Another conclusion due to current study results was that introduction of new RN was limited and why organisational support are highlighted by the participants. According to shortage of RN, introduction was described as a burden for RN with already high working load in their day-to-day work. Therefore, organisational support by department of human relation (HR) could be of great help though the hospital has continually ongoing recruitment due to shortage of RN [26, 27]. High demands such as emergency medical procedures (high number of severe ill patients) in relation to low level of control (number of beds, patient/RN, routines) increase RNs working load why organisational support are stressed, and therefore nurse preceptorship is one way to relieve RN’s workload [28]. RN’s task is to conduct high quality of nursing towards an improved working environment without focus on repeated introduction of new RN. By work shifting general and repetitive tasks such as IT-system, rules and regulation at the hospital etc. (from RN to HR-department), RN working environment could be improve; doing what RN are educated for, caring for patients [1, 3, 25]. According to limited organisational possibilities, improvement towards healthy workplaces by creating balance between demands (organisational, private) and control of daily work, could decrease RN ethical stress [28]. Moreover, organisational support could facilitate teamwork towards patient safety instead of feeling as being a tile in a box without possibilities to influence how work are conducted, i.e. having greater say in the day-to-day work; nursing.

Introduction could be divided into two parts: one general conducted by HR-department (laws, salary, personal administrative tasks) and one specific part conducted by specific RN preceptors at the clinic (nursing tasks at the care unit). Organisational support [8] by HR-department offers a centralize and general introduction (two days) for all new RN at the hospital level in the beginning of every month due to needs within the recruitment processes of RN. Thereafter, follows specific information due to the care unit/specific department, performed by specific RN preceptors employed for example at the internal medicine department, grounded in nursing conducted by expert RN with specific tasks as introduction [24]. A qualitative planned and structured introduction is one suggestion to provide opportunities to be person-centredness [4, 13, 14] due to new RNs need and wishes (voicing their concerns) to improve patient safety [1] within a healthy working environment balancing work and private demands [28].

Moreover, research [4, 5, 29, 30] show that managers affect RNs working environment, and why the significant of leadership for good work environments is stressed, such as relationship-oriented shared leadership [11]. However, the results highlight that leadership, first- and second-line managers, was not always present and available at the care unit [6, 7]. Though leadership is a key factor for sustainable working life, especially in turbulent care settings with high staff mobility and high workload [10], improvement of managerial communication areas is stressed to improve RNs working environment for patient safety. One suggestion to support daily work is create “learning rooms” at the care unit where RN could post questions on a wall or easily find and read about guidelines, routines, research relevant for the specific care units [1, 3]. Posted questions is then handled by first line managers and/or team leaders and/or nursing preceptors facilitate safer workplace culture and one where RN are able to easily voice their concerns due to day-to-day of their workplaces [25, 28]. However, to address visible leadership to support RN working environment. leaders need to be present and available for daily chat around added nursing questions, i.e. professional relationship by supportive leadership [24, 26, 31]. Another suggestion is that second manager (meso, department level) visit the care unit once per month at a regular staff meeting to visualize leadership due to nursing at the specific care unit for improved professional development by commitment in nursing. Collaboration (expert to expert) with all involved is known as person-centred approach based on RN as capable persons (resources, abilities) together in partnership with managers, staff members and patients (teamwork) improve healthcare within a safe and healthy working environment [14, 24, 31–33]. Though a person is more than her/his education/experiences of healthcare, a RN as well as staff members or patients, is persons with resources, abilities, needs and wishes. Therefore, supported person-centred leadership [9] improve RNs working environment by creating partnership (first line manager, individual RN, patients) towards a healthy work condition due to high patient safety [32, 33]. Above person-centred approach [4, 14, 33] RN need organizational commitment [8, 9] at work to stay in nursing [3]. Improved working environment include balance of workload according to supported managerial work using person-centred approach to manage complexed nursing due to severe ill patients in a hospital setting [13, 28, 33].

### Implication for research and practice

RN’s experiences of high demands (emergency medical procedures) and low level of control (hospital aspects), increase RNs working load, therefore managerial support is stressed to facilitate complexed nursing within a healthy working environment. One suggestion to improve RNs working environment is implementing person-centred work, a partnership where experts (managers, health professionals, patients, relatives) collaborate as a team creating committed caring and working environment. By creating communication areas such as learning rooms, staffed with IT-oriented staff, who support RNs IT-related tasks (computers, medical records, forms, work tools), as well as nursing preceptors who support with relevant guidelines, routines and research in nursing. Another suggestion is conducting journal clubs once per month for improved evidence-based nursing as well as structural carrier planning for each individual RN toward professional development in nursing. Above improvement aims to prevent unnecessary obstacles in daily work towards high quality of care within a healthy working environment.

### Limitations

There are some limitations with current study. The sample (n = 38/55) of registered nurses who had leave their work at two care units (internal medicine, emergency care) at one university hospital in western part of Sweden is limited due to drop out (n = 17), limited workplaces (internal medicine wards, emergency department) within one university hospital settings, and why national and international future research is needed to verify results from current study. Above sample is dependent to its context (university hospital in Sweden internal medicine, emergency care) therefore, conclusions regarding RN’ reason to end their duty are limited and why our results should be considered above limitations. Moreover, interviews were performed by distance (verbal communication, telephone, video) which could influence on the quality of interviews due to limited responsiveness (lack of face-to face interaction). However, trustworthy is described due to number of included participants (*n* = 38) and their variation of age, employment and training in nursing.

According to that participant had finished their work at the hospital without loyalties as employees at the university hospital as well as no personal relationship between participants and interviewees (HR-department) were described, strengthen the results due to lack of asymmetries. Participant could freely describe his/her experiences (semi-structured questions) without risk of personal discredits at the workplace. Data was first analysed individually by the two authors, following a well-used scientific systematic methodology such as manifest qualitative content analysis [18, 19]. Thereafter, analysis was conducted until agreement was reached among the researchers without personal relationship to participants, managing individual preunderstanding due to trustworthiness. According to credibility, data analysis is illustrated by examples (Table 1) and quotes are used within the results. Summary, current study are grounded in ethical consideration such as voluntarily participation and confidentiality was assured (results are presented in groups without personal identification) to safeguard that participant did not got harmed [24], as well as ethical points of view due to person-centredness [13, 14]. However, study limitations such as sample (few departments at one hospital) and interviews performed by distance (lack face-to-face), points out that future studies are needed to confirm the results.

## Conclusions

The results showed the significant of RN’s working environment to create commitment at work focusing balance between work and personal life. Organisational support and person-centred leadership, grounded in relationship (collaboration, shared decision-making), conducted by first line managers are key factors to improve healthy working environment. First line managers who are responsible for RNs working environment are key persons to decrease shortage of RN in healthcare by offering healthy working environment. To improve RNs working satisfaction, balance between work and private life is significant to improve by supportive organisational and leadership commitment at work [32, 33]. A person-centred approach, expert (manager) to expert (RN) built on shared decision-making in partnership are one way to develop organisational commitment towards a healthy working environment where work and private life are balanced due to demand and control. High demands (emergency medical procedures) and low level of control (hospital aspects) increase RNs workload, and why managerial support are stressed to facilitate nursing within a satisfactory working environment [28]. Satisfaction at work is, among other things, grounded in professional development in nursing for patient safety. Therefore, career planning for RN is needed supported at both micro (specific care units) and meso level (central hospital settings). Organisational support facilitates nursing, for example by structured duty schedule and standardized introduction of new staff members through the hospital settings (micro-meso level). Above developed managerial pathways (organisational, leadership) improve and relieve high workload, one key stone to reduce turnover of RN.

In order to justify the results from current qualitative study, European standard for person-centred care and Karasek and Theorells Job Demand-Control Model are tools to explain the relationship between job characteristics and RN health and well-being. The conclusions are that partnership as well as job demands and job control are key factors that influence RNs working environment in relation to limited organizational support, lack of visible leadership and limited healthy working environment [28, 33].

Another way could be to conduct future quantitative research to examine and analyze the relationships by using Structural Equation Modeling (SEM), a versatile method used in social and natural sciences that combines elements of both regression analysis and factor analysis to understand complex relationships among multiple variables simultaneously, for example factors that influence RNs working environment [34].

## Data Availability

We do confirm that all data generated or analyzed during this study are included in this published article.
